# Ephrin-A1 Is Up-Regulated by Hypoxia in Cancer Cells and Promotes Angiogenesis of HUVECs through a Coordinated Cross-Talk with eNOS

**DOI:** 10.1371/journal.pone.0074464

**Published:** 2013-09-09

**Authors:** Yong Song, Xiao-Ping Zhao, Kai Song, Zheng-Jun Shang

**Affiliations:** 1 The State Key Laboratory Breeding Base of Basic Science of Stomatology (Hubei-MOST) and Key Laboratory for Oral Biomedicine Ministry of Education, Wuhan University, Wuhan, China; 2 Department of Oral and Maxillofacial-Head and Neck Oncology, School and Hospital of Stomatology, Wuhan University, Wuhan, China; Seoul National University, Republic of Korea

## Abstract

Hypoxia, ephrin-A1 and endothelial nitric oxide synthase (eNOS) have been proved to play critical roles in tumor angiogenesis. However, how ephrin-A1 is regulated by hypoxia and whether ephrin-A1 cooperates with eNOS in modulation of angiogenesis remain to be addressed in details. Here we demonstrated that both ephrin-A1 in squamous cell carcinoma cells (SCC-9) and especially soluble ephrin-A1 in the supernatants were up-regulated under hypoxic condition. An increased nitric oxide (NO) production in human umbilical vein endothelial cells (HUVECs) was observed in ephrin-A1-induced angiogenesis which was reversed after co-culture with eNOS specific inhibitor, N-nitro-L-arginine methyl ester hydrochloride (L-NAME). Western blot analysis confirmed that both phosphorylation of Akt_Ser473_ and eNOS_Ser1177_ were up-regulated in ephrin-A1-stimulated HUVECs, with the total eNOS expression unchanged. The specific inhibitor of phosphatidylinositol 3-kinase (PI3K), LY294002, significantly down-regulated ephrin-A1-induced expression of phosphorylated Akt_Ser473_ as well as phosphorylation of eNOS_Ser1177_. These results revealed a possible novel mechanism whereby ephrin-A1 is regulated in tumor microenvironment and promotes angiogenesis through a coordinated cross-talk with PI3K/Akt-dependent eNOS activation which may relate to normal vascular development and tumor neovascularization.

## Introduction

A variety of pro-angiogenic and anti-angiogenic factors participate in controlling angiogenesis which plays essential roles in tumor growth and metastasis [1]–[3]. Ephrin-A1 and its primary receptor, EphA2, are not only expressed in multiple malignancies, but also play a vital role in normal angiogenesis and tumor neovascularization [4]. Over-expression of ephrin-A1 in tumor cells can promote the angiogenic process, while knock down of ephrin-A1 in tumor cells contributes significantly to reduction of tumor-induced endothelial cell migration in vitro and microvascular density in vivo [5]. Our previous study showed that over-expressed EphA2 may contribute to tumor angiogenesis and have prognostic value in tongue carcinoma [6]. There is sufficient experimental evidence suggesting that activation of EphA2 on endothelial cells (ECs) is required for ephrin-A1 to exert its angiogenic effects in vitro and in vivo [7]. However, the regulating factors and mechanisms by which ephrin-A1/EphA2 promote tumor angiogenesis were not well clarified.

It has been reported that several growth factors and cytokines may induce the expression of ephrin ligands, such as tumor necrosis factor-α (TNF-α), interleukin-1β (IL-1β), et al [8]. Hypoxia is one of the most common and important features in tumor microenvironment, and contributes to induction of various angiogenic factors [9]. Recently, HIF-1α, a hypoxia-inducible transcription factor, has been found to up-regulate ephrins and Eph receptors in mouse skin [10]. In head and neck cancers, increased ephrin-A1 expression was associated with pO_2_ in tumor microenvironment [11]. Although ephrin-A1 plays a critical role in tumor angiogenesis and seems to be involved in response to hypoxia, most of previous studies have mainly focused on ephrin-A1 as a membrane-bound protein. To our knowledge, there is relatively little direct evidence whether hypoxia can induce cancer cells to produce ephrin-A1, especially the soluble form, or not.

The mechanisms underlying ephrin-induced angiogenesis have not been fully understood yet. Until now, only a few signaling pathways, such as MAP/ERK and PI3K [12], [13], have been found to be affected by ephrin-A1. Moreover, the promotion as well as the inhibition of the same signaling pathway by ephrin-A1 was observed in different cells or cancer types. It is well-known that eNOS and NO play a critical role in endothelial migration and angiogenesis [14]. Sufficient evidence showed that eNOS is expressed predominantly in tumor vascular endothelial cells, and its production NO acts as direct effector molecule in various angiogenic factors-induced tumor angiogenesis [15], [16]. Therefore, it is not surprised to suppose that eNOS/NO may also mediate ephrin-A1-induced tumor angiogenesis. Unfortunately, no direct information is available on the cross-link between ephrin-A1 and eNOS during modulation of angiogenesis in endothelial cells so far.

The current study investigated the mechanisms underlying ephrin-A1 modulation of angiogenesis through examining the effect of hypoxia on ephrin-A1 expression and secretion in tumor cells and the possible association of ephrin-A1 with eNOS/NO in tumor angiogenesis. Our data confirmed that both ephrin-A1 expression and soluble ephrin-A1 secretion in tumor cells were increased under hypoxia stimulation. The ephrin-A1-induced angiogenesis was accompanied with eNOS phosphorylation and NO production, which was blocked by L-NAME. Further study showed that activation of PI3K/Akt signal pathway is required for the crosstalk between ephrin-A1 and eNOS in promoting angiogenesis. Our results suggested that up-regulated ephrin-A1 in tumor hypoxic microenvironment may promote angiogenesis via PI3K/Akt/eNOS pathway.

## Materials and Methods

### Materials

Recombinant human ephrin-A1-Fc chimera and recombinant human IgG1 Fc were purchased from R&D systems (Minneapolis, MN, USA). Antibodies against EphA2, eNOS and ephrin-A1 were purchased from Santa Cruz Biotechnology Inc. (Santa Cruz, CA, U.S.), Akt and phospho-eNOS (Ser1177) (P-eNOS_Ser1177_) from Cell Signaling Technology (Beverly, CA, U.S.), phospho-Akt (Ser473) (P-Akt_Ser473_) from Epitomics, Inc.(Burlingame, CA, U.S.).

### Cell Culture

SCC-9 cell line, which was purchased from American Type Culture Collection (ATCC, Manassas, VA, U.S.), was kindly provided by Professor Wen-Feng Zhang. The cells were cultured in DMEM/F12 (Hyclone, UT, USA) supplemented with 10% FBS (Gibco, Carlsbad, Calif, USA). U-251 GBM cell line was purchased from China Center For Type Culture Collection (CCTCC, Wuhan, China), which was cultured in MEM (Hyclone, UT, USA) supplemented with 10% FBS (Gibco, Carlsbad, Calif, USA). Primary HUVECs were kindly provided by Professor Yi-Fang Zhao and Dr. Hai-Xiao Zou [17] and cultured in EC basal medium-2 (EBM-2; Lonza, Walkersville, MD) supplemented with 2% fetal bovine serum (FBS) and with EGM-2 growth factor mixture (Lonza). HUVECs used in this study were restricted in passage 4 to passage 6. All cells were cultured at 37°C in an atmosphere containing 5% CO_2_. Our studies were approved by the Committees on Ethics in School and Hospital of Stomatology (Wuhan University, reference number 055/2011).

### Cell Migration Assay

Cell migration was examined using the scratch wound assay. Confluent HUVECs in 24-well plate were starved overnight in 0.1% BSA EBM-2 until a wound was made by using a 200 µl pipette tip. After rinsing with PBS three times to remove the detached cells and cellular debris, growth factor-free medium with ephrin-A1-Fc (1 µg/ml) alone or together with L-NAME (100 µM) or LY294002 (10 µM) was added into each well. Growth factor-free medium with or without recombinant IgG1 Fc (1 µg/ml) was taken as control. Images at the same position along the scratch wound were taken at 0 h, 24 h. The percentage of wound closure at each time point was calculated by the following formula: [1-(current wound area/initial wound area)]×100.

### In vitro Tube Formation Assay

Sub-confluent HUVECs were resuspended in growth factors-free EBM-2 containing ephrin-A1-Fc (1 µg/ml) alone or together with L-NAME (100 µM) or LY294002 (10 µM), and seeded on the pre-solidified BD matrigel (BD Bioscience) in 96-well plate (3×10^4^ cells/well). Plates were then incubated at 37°C, 5% CO_2_ for 6 h. Images were taken from each group. All the branch points of tube structures in each well were counted to quantify the degree of tube formation. Growth factor-free medium with or without recombinant IgG1 Fc (1 µg/ml) was taken as control. The percentage of tube formation was calculated by taking the recombinant IgG1 Fc control group as 100% tube formation. The experiments were repeated at least three times under similar conditions.

### NO Concentration Detection Assay

Total NO concentration in culture medium was detected by measuring the concentration of nitrate and nitrite by modified Griess reaction method. Total Nitric Oxide Assay Kit (Beyotime, China) was used. The optical densities at 540 nm wavelength were recorded using a Micro-plate Reader (Thermo MutliscanMK3; Thermo Fisher Scientific, Waltham, MA, USA) and the concentrations of NO were calculated according to the standard curve.

### Block Assay

Block assay was carried out to evaluate the function of eNOS in ephrin-A1-induced HUVECs angiogenesis and ephrin-A1 effect on P-eNOS_Ser1177_ through PI3K-Akt pathway. L-NAME (100 µM) or LY294002 (50 µM) was added in the culture medium to block eNOS or PI3K. HUVECs of the control groups were cultured in the growth factor-free medium with or without recombinant IgG1 Fc (1 µg/ml).

### Immunofluorescence Assay

HUVECs growing on coverslips were incubated in 2% FBS EBM-2 containing 1 µg/ml ephrin-A1-Fc for 0 h, 8 h and 24 h. Coverslips were washed with PBS and fixed in 4% cold paraformaldehyde for 10 min. Following treatment with 5% BSA-PBS at 37°C for 30 min, cells were incubated with primary rabbit polyclone antibody against eNOS (dilution 1∶600) or EphA2 (dilution 1∶400) at 4°C overnight. Cells were then washed with PBS three times for 15 min and exposed to Alexa Fluor 488-conjugated goat anti-rabbit IgG (dilution 1∶300) for 1 h at 37°C. The nuclei were stained for 2 min in Hoechst (dilution 1∶10000) (Sigma, USA), followed by three further washes in PBS for 15 min. All coverslips were covered with slides and mounted on fluorescence microscope (Leica DM4000B, Germany) for detection. Negative controls were treated in the same procedure but omitting the primary antibody.

### Cell Proliferation Assay

HUVECs in 96-well plate (1×10^3^ in 100 µl/well) were exposed to ephrin-A1-Fc (1 µg/ml) for 0 h, 24 h and 48 h. The cell proliferation rate was measured by using a Cell Counting Kit-8 (Dojindo, Tokyo, Japan) by adding 10 µl WST-8 in each well at indicated times. Absorbance at 450 nm wavelength was recorded using a Micro-plate Reader (Thermo MutliscanMK3; Thermo Fisher Scientific, Waltham, MA, USA).

### Polymerase Chain Reaction

Total RNA was extracted from trial groups. cDNA was synthesized with RevertAid™ Premium First Strand cDNA Synthesis Kit (Fermentas, Glen Burnie, MD, USA). Real-time PCR was carried out using the Maxima™ SYBR Green qPCR Master Mix kit (Fermentas, Glen Burnie, MD, USA) and spectrofluorimetric thermal iCycler1 (Bio-Rad, Hercules, CA). For eNOS, primer sequences are 5′-GTGGCTGTCTGCATGGACCT-3′ (forward) and 5′-CCACGATGGTGACTTTGGCT-3′ (reverse), product size 121 bp. Human glyceraldehyde-3-phosphate dehydrogenase (GAPDH) was taken as an internal control. The mRNA expression level in each group was calculated by the comparative ΔΔCt.

### Hypoxia Experiment

SCC-9 cells were exposed to hypoxic conditions (1% O_2_) or normoxic conditions (21% O_2_) for 0 h, 6 h, 12 h, 24 h and 48 h. Cells were harvested and lysed in SDS sample buffer for Western blot assay. Supernatants were collected and centrifuged at 3,000×g for 10 min to remove the debris. Equal amount of supernatant was loaded to detect the soluble form of ephrin-A1 by Western blot analysis [18].

### Cell Rounding Assay

The supernatants (24 h) above were taken as conditioned medium(CM) and concentrated 6× before cell rounding experiment using 10 kDa MWCO Amicon Ultra Centrifugal filter devices (Millipore). U-251 GBM cells were treated with CM or 1 µg/ml recombinant human ephrin-A1-Fc. Inverted microscope was used to observe cell rounding at 15 min, 30 min, and 2 h after treatment.

### Western Blot Analysis

HUVECs in 90% confluency were treated with ephrin-A1-Fc (1 µg/ml) in 2% FBS EBM-2 for indicated times. Protein was harvested in lysis buffer containing protease inhibitor cocktail and phosphatase inhibitor cocktail (Roche, Germany). Equal amount of protein (30 µg) was loaded and separated in 8% SDS-PAGE. And then, proteins were transferred to polyvinylidene difluoride membrane (Millipore Co., Billerica, MA, U.S.) at 200 mA for 2 h (100 mA, 1 h for ephrin-A1) at 4°C, blocked in TBS containing 5% BSA (w/v) and 0.1% Tween-20 for 1 h, and incubated with appropriate primary antibodies at 4°C overnight. The dilution of antibodies was as follows: ephrin-A1 (1∶500), eNOS (1∶600), P-eNOS_Ser1177_ (1∶1000), Akt (1∶1000), P-Akt_Ser473_ (1∶1000) and β-actin (1∶2000). After five 5 min washes, the blocks were incubated with horseradish peroxide-conjugated secondary antibody (dilution 1∶10,000) (Jackson Immunoresearch Laboratories) for 1 h at room temperature. At last, bands were examined by using ECL plus western blotting detection reagents (Beyotime, China). Western blot analysis was repeated at least three times under similar conditions.

### Statistical Analysis

All data were presented as mean±SEM. One-way analysis of variance (ANOVA) was used to compare the difference among trial groups. *P* values less than 0.05 were considered significantly different.

## Results

### Ephrin-A1 Enhanced Angiogenesis in vitro

EphA2 receptor expresses positively in our cultured HUVECs (Figure S1A–C). The non-pre-clustered human recombinant ephrin-A1-Fc was used to determine the effects of ephrin-A1 on proliferation, migration and tube formation in HUVECs. Our results showed that ephrin-A1-Fc could significantly increase HUVECs migration (Figure S2A, B) and tube formation on matrigel (Figure S3A–D), without affecting on cell proliferation (Figure S4). These results were consistent with previous reports [19], confirming ephrin-A1 modulation of angiogenesis.

### Ephrin-A1 Induction of Angiogenesis is Mediated by eNOS Activation and NO Production

**Figure 1:**
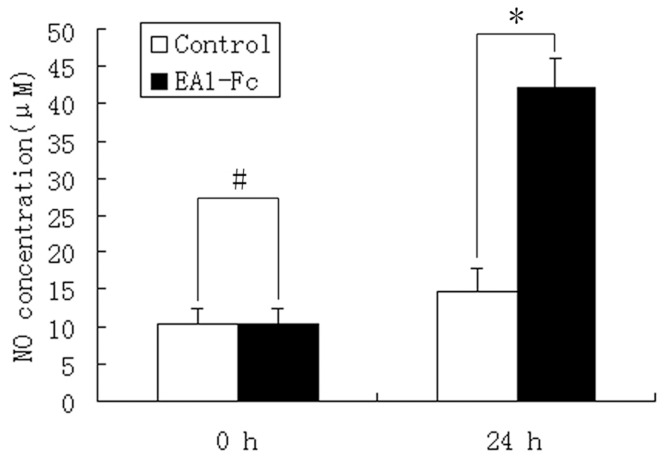
NO production in the supernatants of HUVECs after incubation with ephrin-A1-Fc (1 µg/ml) for 0 h, 24 h. Data showed a significantly increased NO production in the EA1-Fc group. (*, *P*<0.05, n = 3).

**Figure 2:**
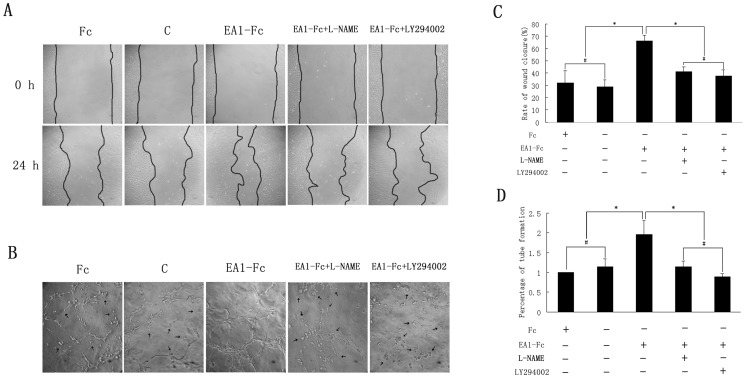
A critical role of eNOS/NO and PI3K/Akt in ephrin-A1-Fc activation of angiogenesis in HUVECs. A: Cell migration assay showed that L-NAME and LY294002 inhibited ephrin-A1-stimulated migration of HUVECs (200×). B: Tube formation assay showed that L-NAME and LY294002 inhibited ephrin-A1-stimulated tube formation of HUVECs (200×). Arrowheads presented the HUVECs that stretched insufficiently. C: Quantification of A. D: Quantification of B. (*, *P*<0.05, n = 3).

Although eNOS/NO have been considered as a vital effector in tumor angiogenesis, no previous studies have addressed whether eNOS/NO mediate ephrin-A1-induced angiogenesis. NO detection assay showed a dramatic increase of NO production in the supernatant of cultured HUVECs after incubation with 1 µg/ml ephrin-A1-Fc for 24 h (Figure 1). There was a significant statistical difference in NO production between control group (10.4±3.4 µM) and ephrin-A1-Fc-stimulation group (42.1±4.1 µM). When NO production was inhibited with L-NAME (100 µM), wound closure rate was suppressed dramatically (Figure 2A) (Figure S2A, B), and less intact tube structures and branch points were observed in ephrin-A1-stimulated HUVECs (Figure 2B) (Figure S3A–D), showing a significant decrease in cell migration and tube formation (Figure 2C, 2D). These results suggested that eNOS/NO may play a key role in ephrin-A1-induced angiogenic process.

**Figure 3:**
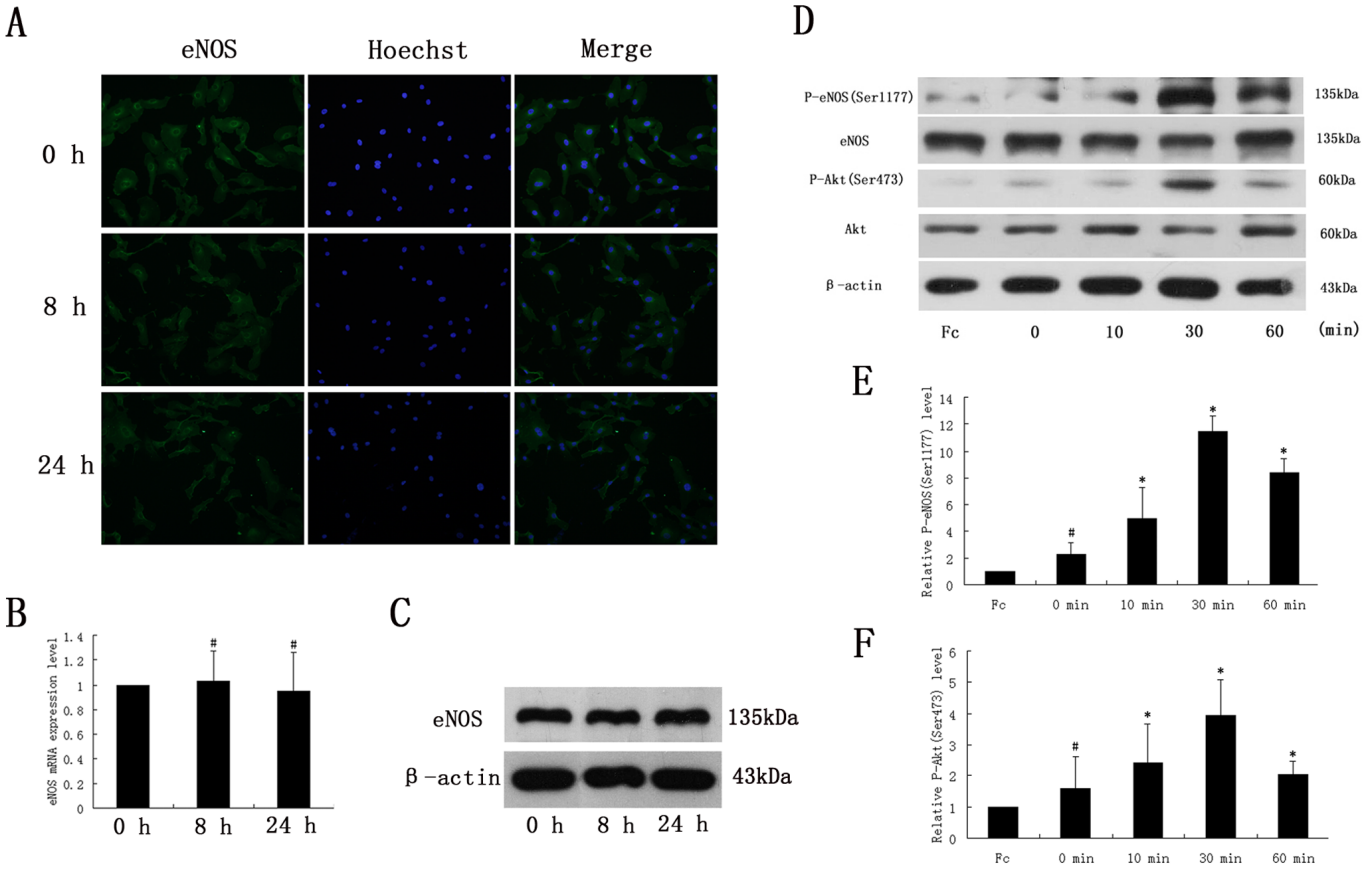
Effect of ephrin-A1-Fc on expression and phosphorylation of eNOS and Akt in HUVECs. HUVECs were exposed to ephrin-A1-Fc (1 µg/ml) for 0 h, 8 h, 24 h. Immunofluorescence (A), Real-time PCR (B) and Western blot analysis (C) demonstrated that ephrin-A1-Fc had no effect on eNOS expression of HUVECs (#, *P*>0.05, n = 3). D: Western blots demonstrating that P-eNOS_Ser1177_ and P-Akt_Ser473_ were up-regulated under ephrin-A1-Fc stimulation in a time-dependent manner. E: Quantity analysis of P-eNOS_Ser1177_. F: Quantity analysis of P-Akt_Ser473._ (*, *P*<0.05, n = 4).

For further study, eNOS expression and phosphorylation status were examined in ephrin-A1-stimulated HUVECs. As shown in Figure 3A, 3B, 3C, no significant eNOS expression changes were detected by immunofluorescence, real-time PCR and western blot analysis. However, ephrin-A1-Fc stimulation caused a rapid time-dependent increase in eNOS phosphorylation at site Ser1177 (Figure 3D) (Figure S5). P-eNOS_Ser1177_ started to elevate significantly in about 10 min and reached to the maximum effect in 30 min and decreased thereafter (Figure 3E). These data suggested that P-eNOS_Ser1177_ activation but not increase of eNOS expression is mainly responsible for the elevated NO production in ephrin-A1-induced angiogenesis.

### PI3K/Akt is Required for ephrin-A1-induced P-eNOS_Ser1177_ in HUVECs

**Figure 4:**
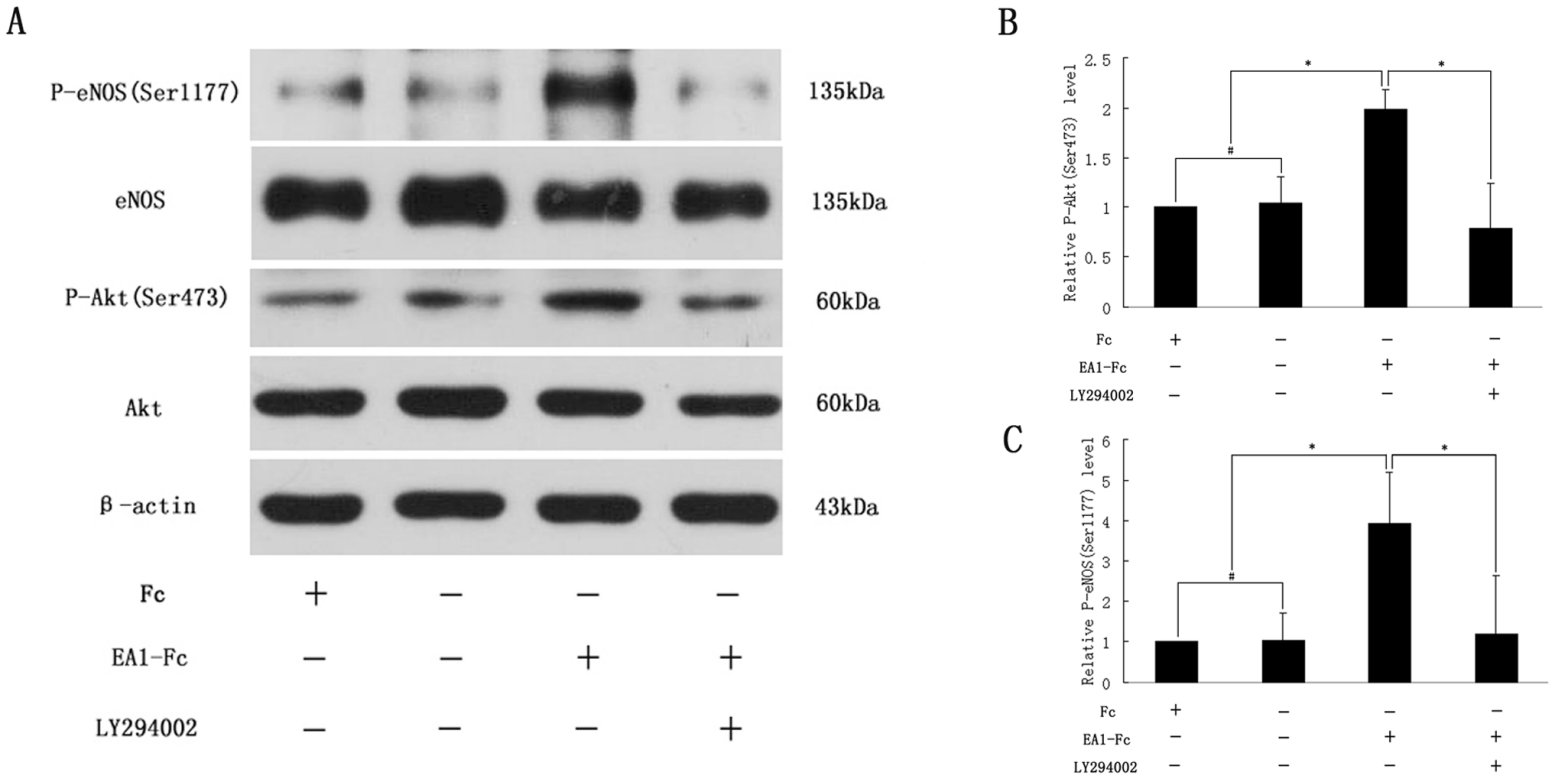
PI3K/Akt mediated ephrin-A1-induced P-eNOS_Ser1177_ in HUVECs. A: Representative Western blots for P-eNOS_Ser1177_ and P-Akt_Ser473_ from HUVECs that were starved in 0.1%BSA EBM-2 overnight and stimulated with ephrin-A1-Fc (1 µg/ml) for 30 min alone or together pre-treated with LY294002. B: Quantity analysis of P-Akt_Ser473_. C: Quantity analysis of P-eNOS_Ser1177_. (*, *P*<0.05, n = 4) (#, *P*>0.05, n = 4).

As shown in Figure 3D, 3F, ephrin-A1-dependent P-eNOS_Ser1177_ was also accompanied by a similar rapid phosphorylation and activation of Akt_Ser473_, suggesting Akt may act as an important upstream modulator that contributes to P-eNOS_Ser1177_ in this process. While PI3K has been taken as a direct controller of Akt [20]–[22], LY294002 (50 µM) was applied in our study to confirm its effect on the phosphorylation of Akt and eNOS. Our results showed that not only P-Akt_Ser473_ but also P-eNOS_Ser1177_ was attenuated by treatment with LY294002 (Figure 4A–C) (Figure S6), uncovering that PI3K is fundamental to Akt phosphorylation by ephrin-A1 and in turn phosphorylates eNOS at site Ser1177. Furthermore, in order to determine whether PI3K/Akt activation was involved in ephrin-A1-induced cellular functions, LY294002 was added into the culture medium and its effects on ECs were evaluated by scratch wound assay and tube formation assay. As shown in Figure 2A, 2B, after exposure to LY294002, the wound closure rate was decreased significantly in comparison with the EA1-Fc group (Figure 2C); HUVECs on matrigel stretched insufficiently, less branch points were recorded and fewer intact tube structures were observed in EA1-Fc+LY294002 group (Fig. 2D). These findings suggested that Akt phosphorylation play a critical role in ephrin-A1 induced HUVECs angiogenic functions. Taken together, we could draw a conclusion that ephrin-A1 induces the activation of eNOS via the PI3K/Akt-dependent signal pathway in its pro-angiogenic functions.

### Hypoxia Up-regulated ephrin-A1 Expression and Soluble ephrin-A1 Secretion in Cancer Cells

**Figure 5:**
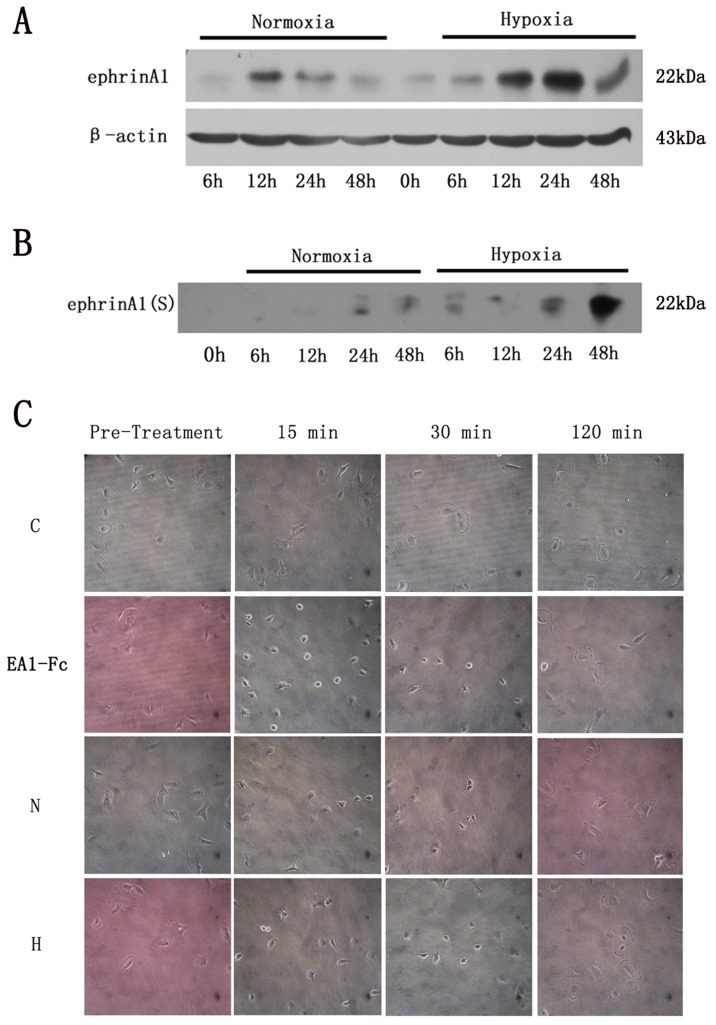
Hypoxia up-regulated ephrin-A1 expression and soluble ephrin-A1 secretion in cancer cells. A: Western blots demonstrating that hypoxia elevated membrane bound ephrin-A1 expression in SCC-9 cells. B: Western blots demonstrating that hypoxia up-regulated soluble ephrin-A1 in supernatants of SCC-9 cells. SCC-9 cell density at 70–80% confluence was taken as 0 h when fresh culture medium was added. C: Cell rounding assay demonstrating that soluble ephrin-A1 in CM could activate EphA2 in U-251 GBM cells. Ephrin-A1(S), soluble ephrin-A1; N, normoxia conditioned medium group; H, hypoxia conditioned medium group.

Cancer cells were cultured under hypoxia and normoxia conditions. Cells and supernatants were harvested for ephrin-A1 analysis by Western blot. Compared with cells cultured in normoxia, SCC-9 cells exposed to hypoxia were detected a marked increase in ephrin-A1 expression in a time-dependent manner, with the maximum effect appearing at 24 h (Figure 5A) (Figure S7A, B). More importantly, ephrin-A1 protein was also detected positively and increased significantly in the supernatants from hypoxia groups, the level of which was much higher in comparison with the normoxia groups (Figure 5B) (Figure S7C, D), suggesting that cancer cells may also secrete soluble form of ephrin-A1 in tumor hypoxic microenvironment. Intriguingly, as shown in Figure 5A, both normoxia and hypoxia group had similar ephrin-A1 expression tendency in SCC-9 cells. The ephrin-A1 started to increase in about 6 h, arrived at its maximum point in 12 h to 24 h and then decreased later. A possible negative feedback mechanism might have been involved in this special process. Ephrin-induced receptor endocytosis has been studied in a number of biological systems. Upon interaction of ephrin-A1 ligand and EphA2 receptor, ligand-receptor complexes can be internalized bi-directionally in tumor cells [23], [24]. This ligand-receptor complexes internalization may further act as a negative feedback motivation in controlling ephrin-A1 expression. So it is possible that the increasing EphA2 activation by both membrane-bound and soluble ephrin-A1 in SCC-9 cells had resulted in the down-regulation of ephrin-A1. As for the reason why ephrin-A1 is increased dramatically between time points 6 h and 12 h even in normoxia group, it still needs further investigation.

Cell rounding is a characteristic response to functional ephrin-A1 [18], [25]. U-251 GBM cells naturally over-express EphA2 and have very low level of ephrin-A1 [26]. The ability of soluble ephrin-A1 in CM to induce cell rounding of U-251 GBM cells was investigated. As shown in Figure 5C (Figure S8), treatment of U-251 GBM cells with either CM or ephrin-A1-Fc resulted in a drastic change in cell morphology, reflecting by cell rounding as early as 15 min, arriving at the peek effect in 30 min and diminishing by 2 h after CM and ephrin-A1-Fc treatment. Although morphological changes of U-251 cells were observed under CM stimulation, further functional experiments are still necessary to demonstrate whether hypoxia-induced soluble ephrin-A1 can induce EphA2 phosphorylation or even tumor angiogenesis.

**Figure 6:**
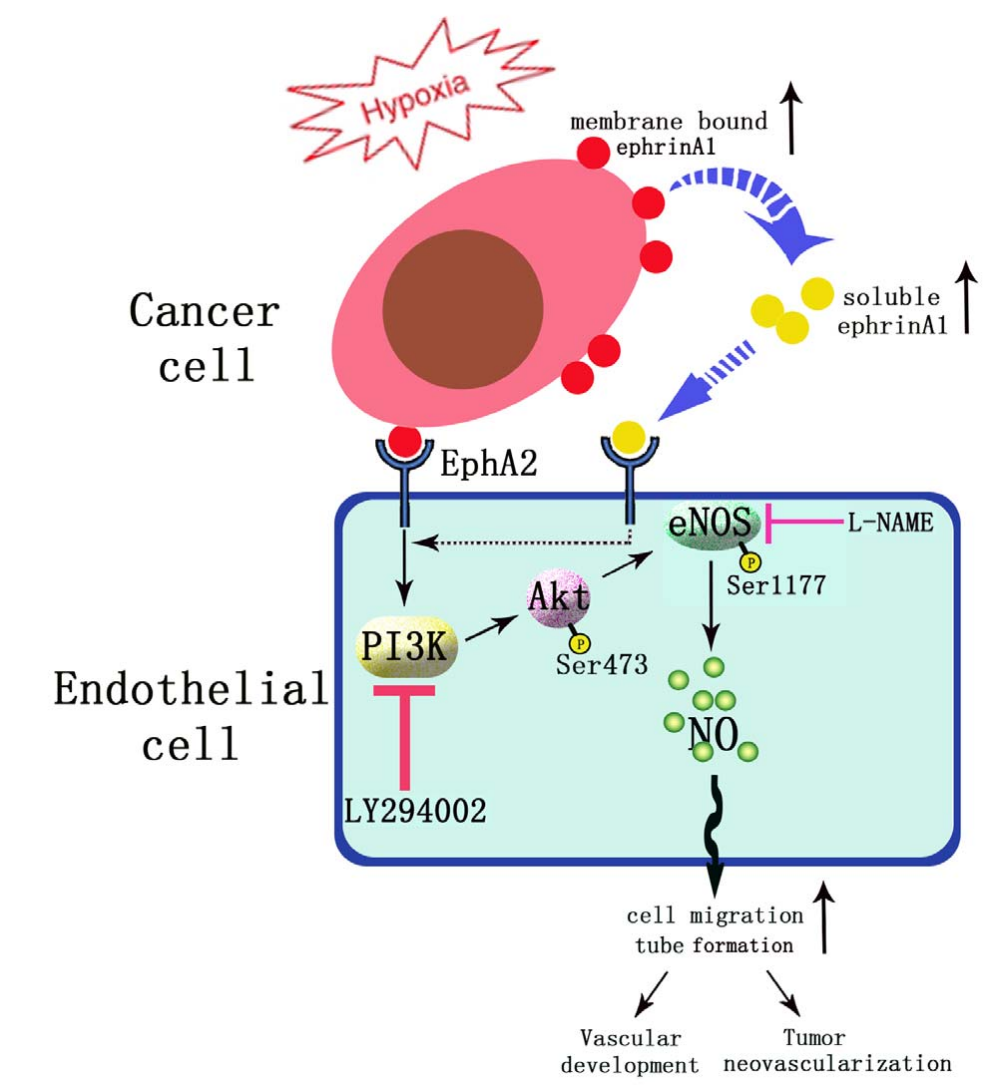
Schematic representation of signaling cascade involved in hypoxia-inducible ephrin-A1 modulation of angiogenesis in tumor hypoxic microenvironment.

The signaling cascade involved in ephrin-A1-induced angiogenesis is characterized schematically in Figure 6.

## Discussion

The current study revealed a mechanism whereby ephrin-A1 modulates angiogenesis and a driving factor for ephrin-A1 up-regulation. Ephrin-A1-induced angiogenesis involves an increased NO production that is subsequent to a PI3K/Akt-dependent activation of P-eNOS_Ser1177_ in endothelial cells. Hypoxia could actively induce enhancement of both membrane-bound and soluble ephrin-A1 in tumor cells. These results provide the first evidence that PI3K/Akt/eNOS signaling cascade may represent a common pathway for hypoxia-inducible ephrin-A1-dependent angiogenesis in vascular development and tumor progression.

Accumulating evidence confirmed that effective angiogenesis requires bioactive NO synthesized by eNOS which is predominantly expressed in vascular endothelial cells. It has been reported that NO is a critical mediator of VEGF-induced angiogenesis which can be blocked by L-NAME [27]. Similarly, Statins can up-regulate eNOS expression, and promote NO-dependent angiogenesis by reducing caveolin-1 abundance [28]. For the first time, we found that NO production is also essential for angiogenesis in response to the pro-angiogenic molecule ephrin-A1. Ephrin-A1-Fc-induced cell migration and capillary assembly were attenuated when ECs were treated with L-NAME. Intriguingly, our further study showed that elevated NO synthesis in ephrin-A1-induced ECs was mainly attributed to eNOS phosphorylation at Ser1177 residue but not enhancement of eNOS expression. It is well known that phosphorylation is one of the most important post-transitional regulatory mechanisms underlying eNOS activation [20], [29]. As supporting evidence of our finding, many kinds of stimuli that promote eNOS activation are also observed to cause phosphorylation at different sites, such as Thr495, Ser633 and Ser1177 [30]. Our findings provide the first evidence that ephrin-A1 has a coordinated cross-talk with eNOS/NO in promoting angiogenesis.

Although the correlation of increased eNOS activity with ephrin-A1-stimulated angiogenesis was found in present study, the signal pathway involved in ephrin-A1-induced enhancement of eNOS activity still needs further investigation. It has been reported that shear stress-induced NO production seems to be controlled by the Akt-dependent phosphorylation of eNOS in cultured ECs [31]. Additionally, Akt protein kinase has been shown to function as an EC survival factor and to promote the tube formation in vitro [32]. Previous studies have also suggested that PI3K/Akt pathway mediate angiogenesis induced by several angiogenic stimuli such as VEGF [33], forskolin [34], microgravity [35], and so on. To investigate the functional involvement of PI3K/Akt pathway in ephrin-A1-eNOS cross-talk, the effect of the LY294002 on ephrin-A1-induced signaling events was determined. After stimulation with ephrin-A1-Fc for 30 min, the phosphorylation of both Akt and eNOS was elevated significantly. This elevation was due to the phosphorylation of Akt_Ser473_ and eNOS_Ser1177_, as demonstrated by western blot analysis. Exposure to LY294002 prevented ephrin-A1-induced expression of P-Akt_Ser473_ and P-eNOS_Ser1177_ apparently, indicating that PI3K/Akt -dependent signal pathway participated in controlling the ephrin-A1-mediated activation of eNOS.

Hypoxia is one of the most important characteristics in tumor microenvironment, and modulates varieties of angiogenic factors such as vascular endothelial growth factor (VEGF) [36]. Ephrin-A1 is also known as an angiogenic factor, and plays pivotal roles in neovascularization in various cancers [4]. Previous studies have found that the ephrin-A1 gene can be induced by tumor necrosis factor-α (TNF-α) in endothelial cells [37]. Recently, Uemura et al. identified ephrin-A1 as a possible candidate hypoxia-inducible gene by microarray analysis of tissue samples from metastatic colorectal cancer [38]. Also, the expression of ephrin-A1 and related genes were markedly reduced in HIF-2α knockdown (kd/kd) endothelial cells, implying the role of hypoxia in ephrin-A1 regulation [39]. In present study, we provided direct evidence that exposure to hypoxia resulted in elevated ephrin-A1 expression in cancer cells by western blot analysis. Ephrin-A1 was first identified as a GPI-anchored protein that requires membrane binding or clustering/oligomerization for its activation of EphA2 [40]. Therefore, our results suggested that up-regulation of membrane-bound ephrin-A1 induced by hypoxia may promote tumor angiogenesis through interaction with its receptor EphA2 on endothelial cells in tumor microenvironment. In addition, we documented in this study that hypoxia up-regulated a soluble form of ephrin-A1 releasing from cancer cells into extracellular environment. In keeping with our finding, ephrin-A1 has also been detected in the serum of patients with liver carcinoma [41]. Recently, several studies have proved that soluble monomeric ephrin-A1 is a functional ligand for EphA2, and that soluble ephrin-A1 released from HeLa and SK-BR3 cells is necessary for cell growth and transformation [4]. Similar to these studies, we observed morphological changes of U-251 cells under CM stimulation. However, further study is needed to confirm whether the hypoxia-induced soluble ephrin-A1 can functionally interact with EphA2 to initiate angiogenesis.

In summary, the most significant and novel findings presented in this study include that 1) ephrin-A1 cooperates with eNOS in promoting angiogenesis in HUVECs; that 2) cross-talk between ephrin-A1 and eNOS is mediated by PI3K/Akt-dependent pathway; and that 3) hypoxia up-regulates both membrane-bound and secreted ephrin-A1 protein in cancer cells. As only a few studies have focused on the ephrin-A1-triggered downstream molecular events, the results reported here identify PI3K/Akt-dependent eNOS_Ser1177_ phosphorylation as a novel mechanism underlying ephrin-A1modulation of angiogenesis (Figure 6).

## Supporting Information

Figure S1 **Expression of EphA2 receptor in the cultured HUVECs.** Immunofluorescence (**A**), RT-PCR (**B**) and Western blot analysis (**C**) demonstrated positive EphA2 expression in HUVECs.
(TIF)

Figure S2 **Cell migration assay showed that L-NAME and LY294002 inhibited ephrin-A1-stimulated migration of HUVECs (200×).** Endothelial cell migration was measured in HUVECs that had been starved in growth factor-free 0.1%BSA EBM-2 overnight and treated with ephrin-A1-Fc (1 µg/ml) for 24 h.
(TIF)

Figure S3 **Tube formation assay showed that L-NAME and LY294002 inhibited ephrin-A1-stimulated tube formation of HUVECs.** A, B, C: Representative images at 40×. D: Representative images at 200×.
(TIF)

Figure S4 **Endothelial cell proliferation was measured in HUVECs treated with ephrin-A1-Fc (1** **µg/ml) for 0 h, 24 h and 48 h.** There was no statistical significance between the EA1-Fc and Control group. EA1-Fc, ephrin-A1-Fc. (#, *P*>0.05, n = 3).
(TIF)

Figure S5 **Effect of ephrin-A1-Fc on phosphorylation of eNOS and Akt in HUVECs.** A, B, C: Western blots demonstrating that P-eNOS_Ser1177_ and P-Akt_Ser473_ were up-regulated under ephrin-A1-Fc stimulation in a time-dependent manner.
(TIF)

Figure S6 **PI3K/Akt mediated ephrin-A1-induced P-eNOS_Ser1177_ in HUVECs.** A, B: Representative Western blots for P-eNOS_Ser1177_ and P-Akt_Ser473_ from HUVECs that were starved in 0.1%BSA EBM-2 overnight and stimulated with ephrin-A1-Fc (1 µg/ml) for 30 min alone or together pre-treated with LY294002.
(TIF)

Figure S7 **Hypoxia up-regulated ephrin-A1 expression and secretion in cancer cells.** A, B: Western blots demonstrating that hypoxia elevated membrane bound ephrin-A1 expression in SCC-9 cells. C, D: Western blots demonstrating that hypoxia up-regulated soluble ephrin-A1 in supernatants of SCC-9 cells. SCC-9 cell density at 70–80% confluence was taken as 0 h when fresh culture medium was added. Ephrin-A1(S), soluble ephrin-A1.
(TIF)

Figure S8 **Cell rounding assay demonstrating that soluble ephrin-A1 in CM can activate EphA2 in U-251 GBM cells.** EA1-Fc, ephrin-A1-Fc; N, normoxia conditioned medium group; H, hypoxia conditioned medium group.
(TIF)
