# Supplementary figures and images for: Ephrin-A1 Is Up-Regulated by Hypoxia in Cancer Cells and Promotes Angiogenesis of HUVECs through a Coordinated Cross-Talk with eNOS
Source: PLoS One. 2013 Sep 9;8(9):e74464. doi: 10.1371/journal.pone.0074464 (PMC3767678; doi:10.1371/journal.pone.0074464)

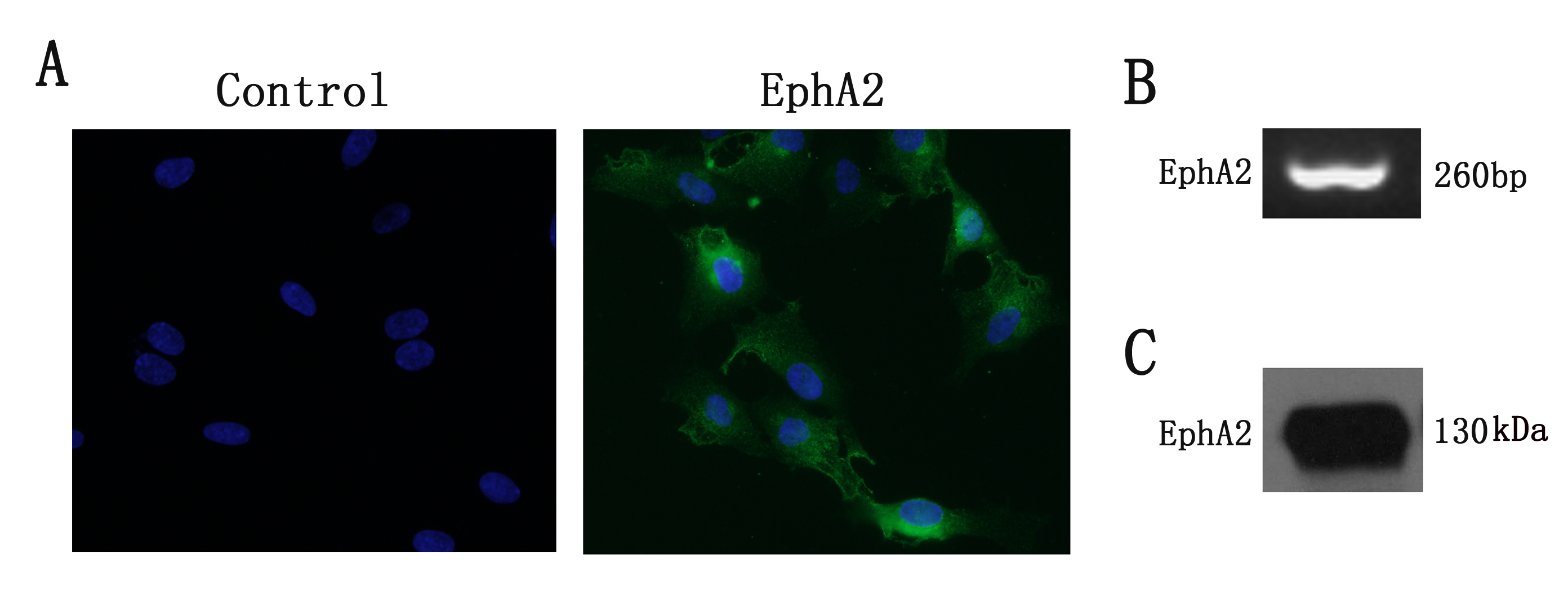

Supplement: Figure S1 — Expression of EphA2 receptor in the cultured HUVECs. Immunofluorescence (A), RT-PCR (B) and Western blot analysis (C) demonstrated positive EphA2 expression in HUVECs. (TIF) [file pone.0074464.s001.tif]

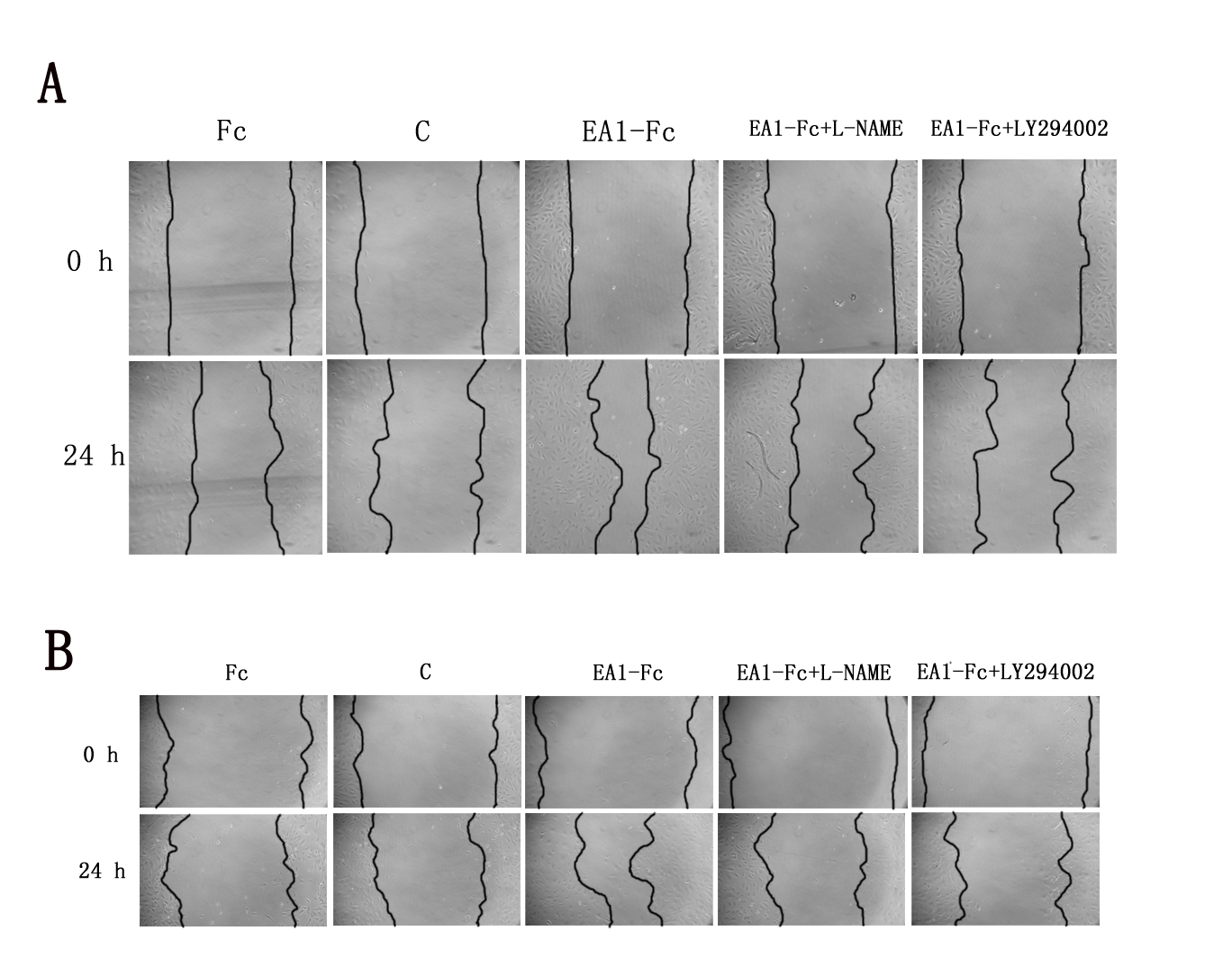

Supplement: Figure S2 — Cell migration assay showed that L-NAME and LY294002 inhibited ephrin-A1-stimulated migration of HUVECs (200×). Endothelial cell migration was measured in HUVECs that had been starved in growth factor-free 0.1%BSA EBM-2 overnight and treated with ephrin-A1-Fc (1 µg/ml) for 24 h. (TIF) [file pone.0074464.s002.tif]

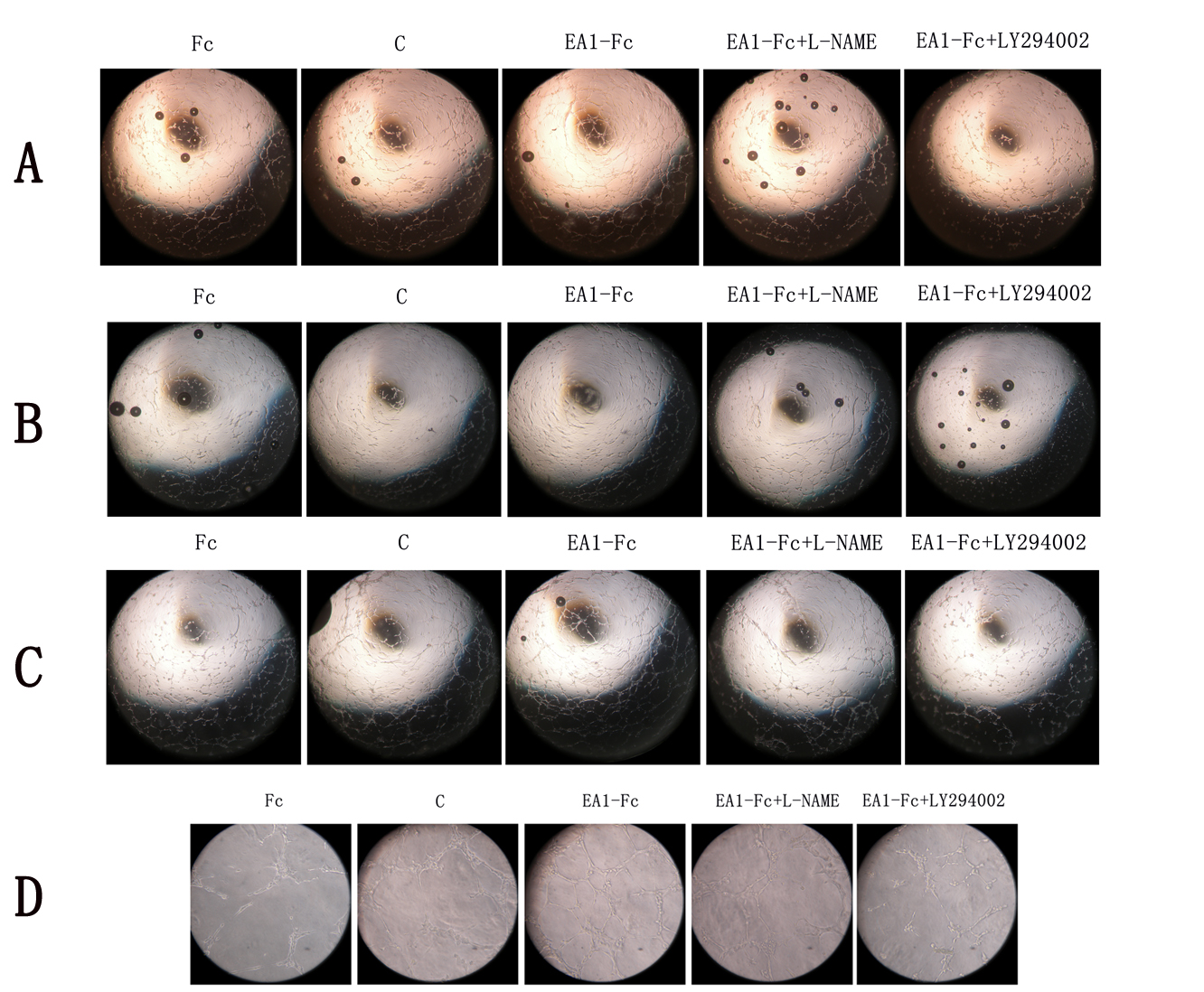

Supplement: Figure S3 — Tube formation assay showed that L-NAME and LY294002 inhibited ephrin-A1-stimulated tube formation of HUVECs. A, B, C: Representative images at 40×. D: Representative images at 200×. (TIF) [file pone.0074464.s003.tif]

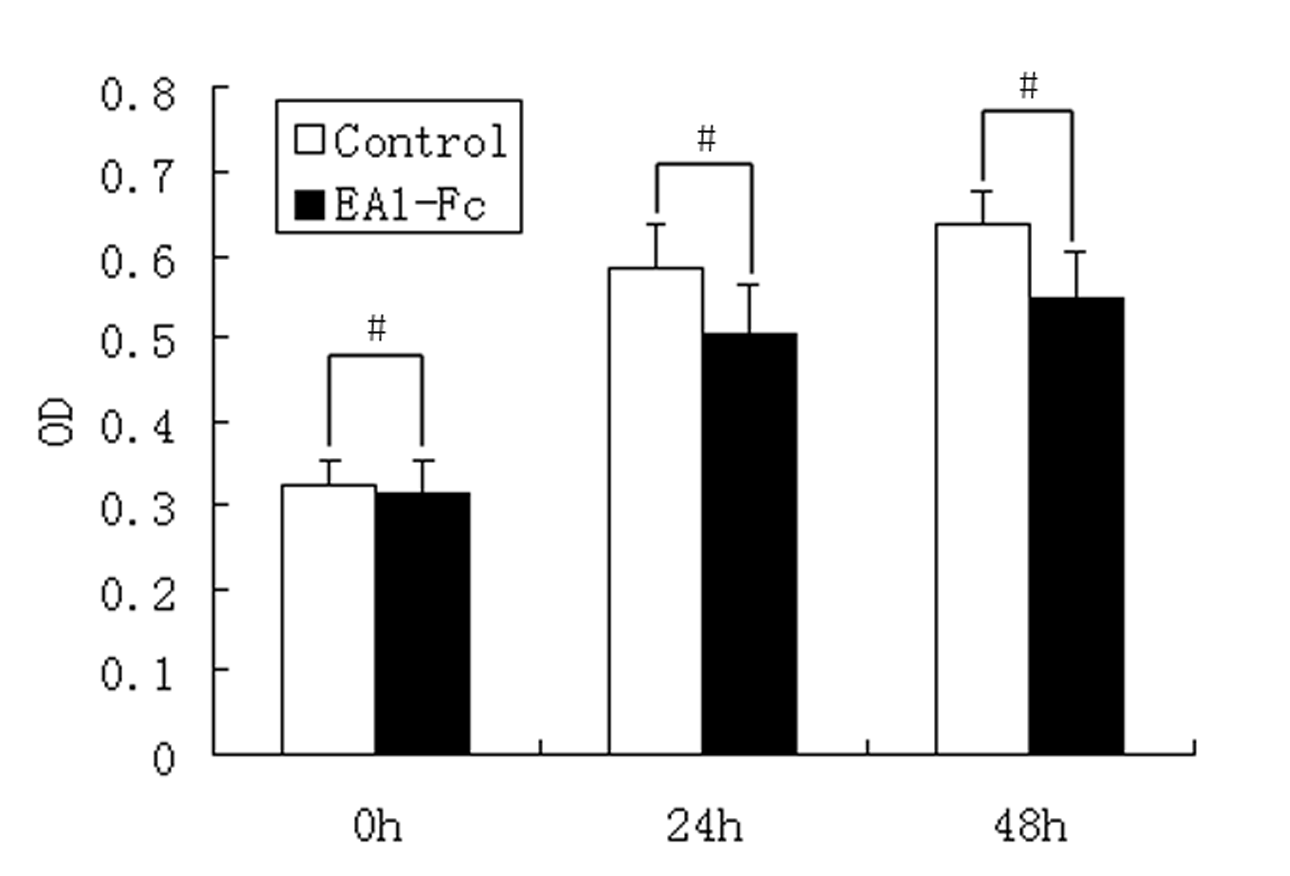

Supplement: Figure S4 — Endothelial cell proliferation was measured in HUVECs treated with ephrin-A1-Fc (1 µg/ml) for 0 h, 24 h and 48 h. There was no statistical significance between the EA1-Fc and Control group. EA1-Fc, ephrin-A1-Fc. (#, P>0.05, n = 3). (TIF) [file pone.0074464.s004.tif]

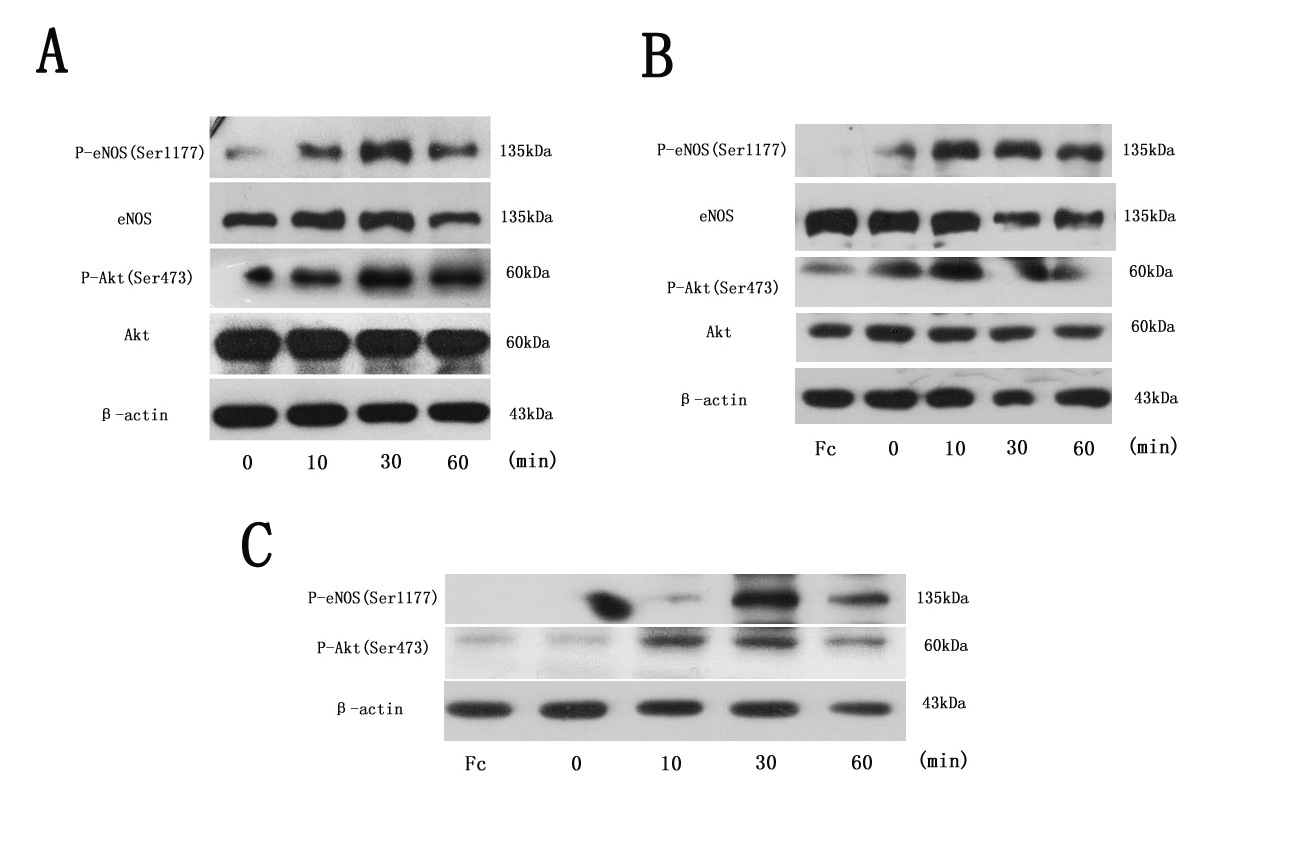

Supplement: Figure S5 — Effect of ephrin-A1-Fc on phosphorylation of eNOS and Akt in HUVECs. A, B, C: Western blots demonstrating that P-eNOSSer1177 and P-AktSer473 were up-regulated under ephrin-A1-Fc stimulation in a time-dependent manner. (TIF) [file pone.0074464.s005.tif]

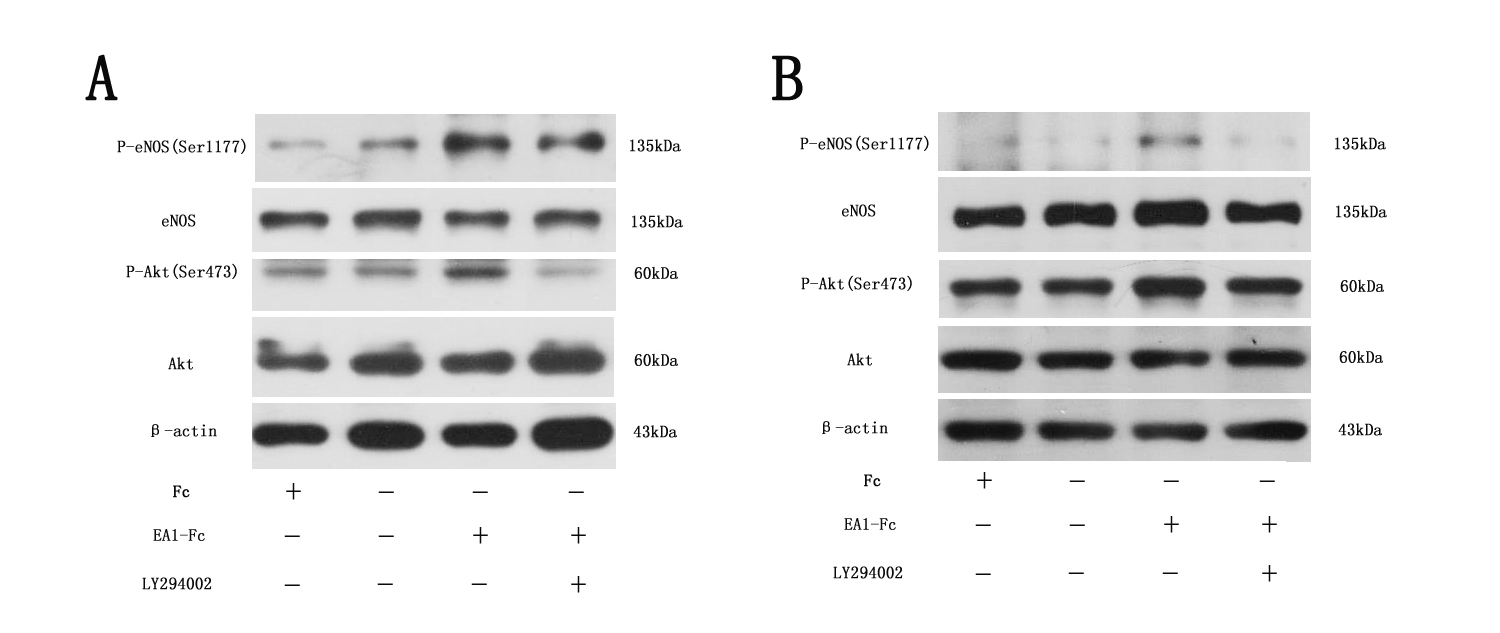

Supplement: Figure S6 — PI3K/Akt mediated ephrin-A1-induced P-eNOSSer1177 in HUVECs. A, B: Representative Western blots for P-eNOSSer1177 and P-AktSer473 from HUVECs that were starved in 0.1%BSA EBM-2 overnight and stimulated with ephrin-A1-Fc (1 µg/ml) for 30 min alone or together pre-treated with LY294002. (TIF) [file pone.0074464.s006.tif]

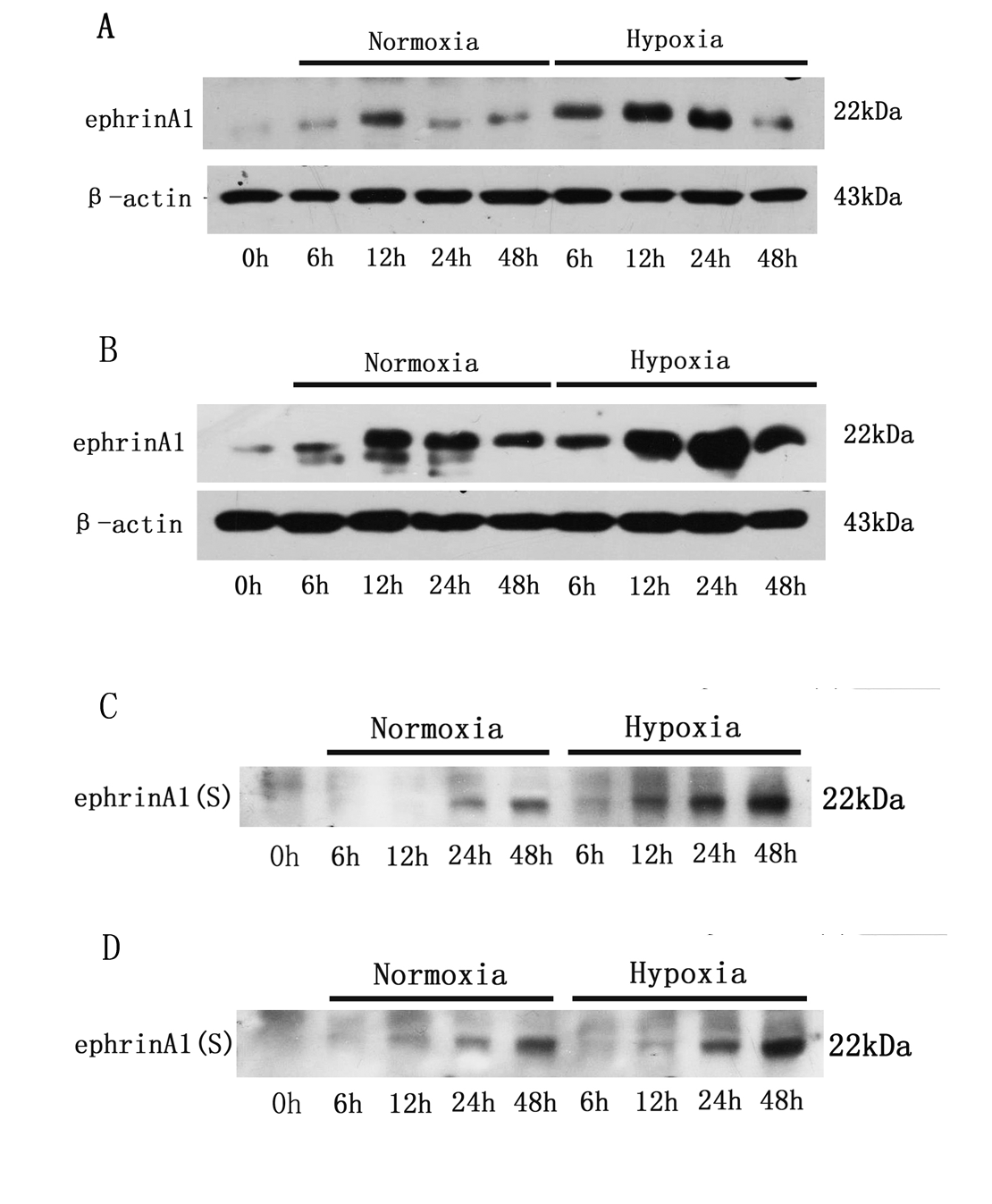

Supplement: Figure S7 — Hypoxia up-regulated ephrin-A1 expression and secretion in cancer cells. A, B: Western blots demonstrating that hypoxia elevated membrane bound ephrin-A1 expression in SCC-9 cells. C, D: Western blots demonstrating that hypoxia up-regulated soluble ephrin-A1 in supernatants of SCC-9 cells. SCC-9 cell density at 70–80% confluence was taken as 0 h when fresh culture medium was added. Ephrin-A1(S), soluble ephrin-A1. (TIF) [file pone.0074464.s007.tif]

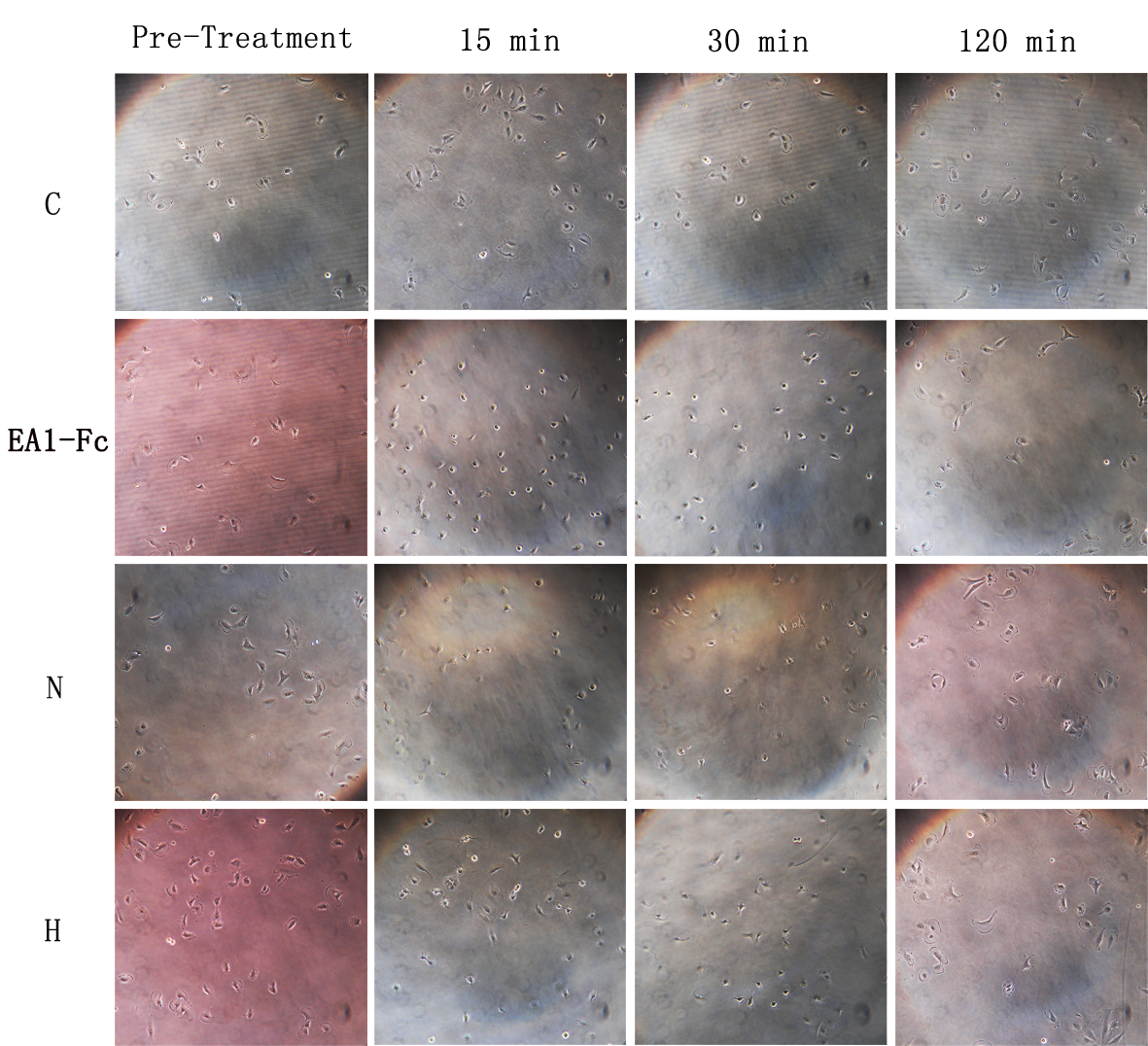

Supplement: Figure S8 — Cell rounding assay demonstrating that soluble ephrin-A1 in CM can activate EphA2 in U-251 GBM cells. EA1-Fc, ephrin-A1-Fc; N, normoxia conditioned medium group; H, hypoxia conditioned medium group. (TIF) [file pone.0074464.s008.tif]
